# Single-trial classification of NIRS signals during emotional induction tasks: towards a corporeal machine interface

**DOI:** 10.1186/1743-0003-6-39

**Published:** 2009-11-09

**Authors:** Kelly Tai, Tom Chau

**Affiliations:** 1 Institute of Biomaterials and Biomedical Engineering, University of Toronto, Toronto, ON, Canada; 2 Bloorview Kids Rehab, Toronto, ON, Canada

## Abstract

**Background:**

Corporeal machine interfaces (CMIs) are one of a few available options for restoring communication and environmental control to those with severe motor impairments. Cognitive processes detectable solely with functional imaging technologies such as near-infrared spectroscopy (NIRS) can potentially provide interfaces requiring less user training than conventional electroencephalography-based CMIs. We hypothesized that visually-cued emotional induction tasks can elicit forehead hemodynamic activity that can be harnessed for a CMI.

**Methods:**

Data were collected from ten able-bodied participants as they performed trials of positively and negatively-emotional induction tasks. A genetic algorithm was employed to select the optimal signal features, classifier, task valence (positive or negative emotional value of the stimulus), recording site, and signal analysis interval length for each participant. We compared the performance of Linear Discriminant Analysis and Support Vector Machine classifiers. The latency of the NIRS hemodynamic response was estimated as the time required for classification accuracy to stabilize.

**Results:**

Baseline and activation sequences were classified offline with accuracies upwards of 75.0%. Feature selection identified common time-domain discriminatory features across participants. Classification performance varied with the length of the input signal, and optimal signal length was found to be feature-dependent. Statistically significant increases in classification accuracy from baseline rates were observed as early as 2.5 s from initial stimulus presentation.

**Conclusion:**

NIRS signals during affective states were shown to be distinguishable from baseline states with classification accuracies significantly above chance levels. Further research with NIRS for corporeal machine interfaces is warranted.

## Background

Access technologies currently available for locked-in individuals are largely limited to corporeal machine interfaces (CMIs), particularly brain-computer interfaces (BCIs) based on electroencephalography (EEG) [1]. EEG has been popular in BCI research owing to its high temporal resolution and non-invasiveness. However, EEG has drawbacks including, but not limited to, its steep learning curve [2], and susceptibility to electrical interference from environmental and physiological sources [3]. Consequently, research efforts have been made towards investigating alternative modalities for brain-computer interfacing. Studies have identified a correlation between cerebral hemodynamic changes - in the form of localized increases in blood flow and oxygen consumption - and electric brain activity [4]. Weiskopf et al. reported on the first BCI based on the blood oxygen level-dependent (BOLD) response measured by functional magnetic resonance imaging (fMRI) [5]. With real-time fMRI feedback, individuals can learn to voluntarily elicit activation in a variety of cortical and subcortical areas [6-8]. Clinical application of a fMRI-BCI is currently impractical due to prohibitive costs and technological limitations [9]. An alternative approach is to measure cerebral and corporeal hemodynamics with near-infrared spectroscopy (NIRS). NIRS is suitable for measuring functional activation in cortical regions 1-3 cm beneath the scalp. The dominant chromophores in the NIR range are oxygenated (HbO) and deoxygenated hemoglobin (Hb), both of which are biologically relevant markers for brain function. Furthermore, water and biological tissue are weak absorbers of light at NIR wavelengths (700-1000 nm) [10]. These factors combine to create an "optical window" through which changes in tissue oxygenation can be monitored. A NIRS instrument consists of light sources by which a tissue volume of interest is irradiated, and detectors that receive light after its interaction with tissue. As a general rule of thumb, light penetration depth is approximately one-half of the distance between a source and a detector [11]. Regardless of penetration distance however, extracerebral blood flow in the superficial tissue typically contributes significantly to NIRS measurements [12].

NIR light undergoes absorption as it penetrates biological tissue; measurements from NIRS instruments yield a response associated with brain activity attributed to this interaction effect. The slow hemodynamic response manifests itself as a small increase in Hb after the onset of neural activity, subsequently followed by a large but delayed increase in HbO peaking at approximately 10 s [13,14] after activation and a corresponding decrease in Hb [15]. Changes in the concentrations of oxygenated (Δ[HbO]) and deoxygenated hemoglobin (Δ[Hb]) can be calculated from changes in detected light intensity using the modified Beer-Lambert Law [11].

Unlike other functional imaging methods, NIRS does not restrict range of motion and has been used to monitor cortical activation in real-world settings [16-18]. NIRS is immune to electrical interference from environmental sources as well as ocular and muscle artifacts [19]. Furthermore, NIRS measurement systems are commercially available at a comparable cost to EEG systems.

Studies on NIRS-BCIs to date have focused on classifying mean amplitude changes in the hemodynamic response induced by mental tasks with well-established psychophysiological bases. Using a 20-channel commercial NIRS measurement system, Sitaram et al. [20] performed offline classification of left-handed/right-handed motor imagery data using amplitude changes in [O_2_Hb] and [HHb] as the class discriminatory features. A maximum accuracy of 89% was achieved using a Hidden Markov Model (HMM). Coyle et al. [21] performed evaluations of a single-channel NIRS system. Able-bodied individuals controlled a binary switch by modulating changes in [O_2_Hb] over the motor cortex and achieved 50-85% accuracy in online trials. Naito et al. [22] investigated the use of high-level cognitive tasks for BCI. Measurements were recorded over the prefrontal cortex with a single-channel, single-wavelength NIRS system. Seventeen locked-in individuals were requested to perform different mental tasks corresponding to 'yes' and 'no' in response to a series of questions. An average offline classification accuracy of 80% was achieved in 40% of the locked-in participants using a non-linear discriminant classifier.

The ultimate goal of a corporeal machine interface is to translate functional intent into a corresponding action. A large body of evidence supports the view that the prefrontal cortex (PFC) plays a central role in cognitive control, the ability to translate thought into action to accomplish a given objective [23]. In particular, functional NIRS (fNIRS) studies have found that changes in affective state generated by emotional induction tasks can elicit activation in the PFC [24-26]. Valenced images have been shown to stimulate changes in prefrontal hemodynamics detectable with NIRS [24]. If emotional induction tasks can consistently generate distinct patterns in the NIRS hemodynamic response, they may be useful in an NIRS corporeal machine interface as a preference detector. In particular, one might be able to use NIRS with nonverbal individuals to distinguish between naturally occurring positive and negative emotional responses to sequentially presented visual stimuli.

Our primary objective was to ascertain the feasibility of using visually-cued emotional induction tasks as a corporeal machine interface mechanism. Several aspects of signal analysis and classification were addressed in realizing this objective, namely 1) artifact removal; 2) feature selection; and 3) classifier selection. The effects of various parameters on classification performance were explored by performing feature selection searches over different task valences, recording sites, and signal analysis window lengths. To our knowledge, this is the first time that feature selection has been used to optimize NIRS signal classification rates. To examine whether or not NIRS data can be represented as linearly separable feature subsets, we compared the offline performance of Linear Discriminant Analysis (LDA) and Support Vector Machines (SVM). Lastly, classification performance was employed as a measure to quantify the latency of the prefrontal hemodynamic response to emotional induction tasks. Note that we use the term corporeal interface to acknowledge that NIRS measurements typically encompass both cortical and superficial tissue blood flow contributions.

## Methods

Ten individuals (5 females, mean age 28.4 ± 6.4 years) participated in the study. Participants had normal or corrected-to-normal vision, and no known indication of the following: 1) degenerative disorders; 2) cardiovascular disorders; 3) metabolic disorders; 4) trauma-induced brain injury; 5) respiratory conditions; 6) drug and alcohol-related conditions; and 7) psychiatric disorders. The aforementioned disorders are known to cause impaired mental function, which may compromise the integrity of collected data. The study was approved by Bloorview Kids Rehab and the University of Toronto Research Ethics Boards. Written consent was obtained from all participants.

### Instrumentation

NIRS measurements were collected with an ISS Imagent (Champaign, IL) functional brain imaging system. Frequency-modulated light at two wavelengths (690 nm and 830 nm) was delivered to the scalp via two-fibre optic bundles ("source pairs") and collected via different fibre-optic bundles ("detectors"). Sources and detectors were held in place with a soft helmet designed to measure over the prefrontal cortex behind the forehead. Its frame, fabricated from a 0.16 cm thick low-density polyethylene, consisted of an adjustable circumference band with a flexible probe overlaying the forehead. Fibres were affixed to the helmet through holes punched in the probe; holes were situated 1.5 cm apart, creating a uniformly spaced grid.

**Figure 1:**
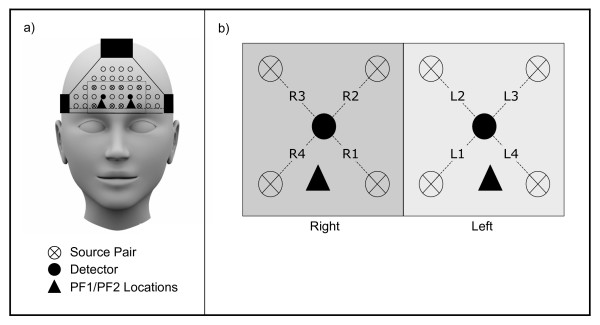
**NIRS probe arrangement**. (a) Sources and detectors were placed symmetrically about the midline in a grid formation, with the inferior row of source pairs positioned along the PF1-PF2 line (International 10/20 Electrode System). (b) Each source pair and its adjacent detector formed one recording site for a total of 8 sites, denoted L1-L4 and R1-R4.

Each side of the prefrontal cortex was interrogated with four pairs of sources and a detector arranged as depicted in Figure 1 for a total of 16 source-detector channels. The arrangement was placed over each participant's frontal lobe with the most anterior row of sources positioned along the PF1-PF2 line (International 10/20 Electrode system [27]). One recording site was formed between each source pair and its adjacent detector. A multiplexer controlled the sequencing of sources such that no two sources were on simultaneously. The time needed to cycle once through all 16 sources was 32 ms, corresponding to a sampling rate of 31.25 Hz.

Source-detector separation distances were fixed at 2.1 cm after preliminary testing on a subset of participants. We quantified the similarity between NIRS signals recorded over 2.1 cm and 3.0 cm, a commonly employed separation distance for fNIRS studies. Signal pairs recorded over the two distances exhibited high correlation values, and it was visually verified that attenuated, but measurable, changes in light attenuation were discernible in signals recorded over 2.1 cm.

Respiration was simultaneously recorded using a piezoelectric respiratory effort belt secured around the participant's chest. Data from this auxiliary transducer were sampled at 60 Hz.

### Protocol

**Figure 2:**
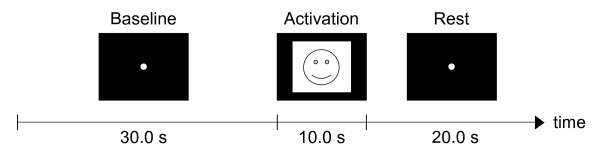
**Sequence of events in a trial**. Sequence of events in a trial. The visual cue is presented for 10 s starting from t = 30 s.

Participants performed trials of an emotional induction task. In a trial, the participant was instructed to rehearse an emotion that he/she associates with the contents of each image for the duration of its presentation. Data collection took place in a dimly lit room. The participant sat in a chair placed approximately 1 m from a LCD monitor and was asked to relax and restrict head movement. A trial consisted of a baseline sequence, a task sequence, and a rest sequence (Fig. 2). Each trial began with a 30 s baseline sequence, during which the participant was instructed to relax and focus his/her gaze on a fixation dot presented at the centre of the screen. The participant then performed the task as prompted on the screen for 10 s. The trial then concluded with a 20 s rest sequence to allow for any activation-induced hemodynamic response to subside. During this post-task rest period, the participant was again instructed to focus on the fixation dot on the screen. Trials were self-paced so that the participant could take short breaks as required.

The participant performed the above emotion induction task in response to 2 stimuli: a pair of valenced images from the International Affective Picture system (IAPS) [28]. Prior to data collection, the participant attended a screening session where he/she performed 5 instances of the emotional induction task for each picture from a stimulus pool of 10 IAPS images. The pool was comprised of 5 images rated for high arousal and positive valence (valence = 7.52 ± 1.53, arousal = 6.37 ± 2.33) and 5 images rated for high arousal and negative valence (valence = 2.94 ± 1.71, arousal = 6.52 ± 2.13). The selected images were IAPS items 8501, 8499, 8080, 8190, 8341, 6313, 1525, 8485, 9622, and 1930. After converting raw light intensity data to changes in attenuation (optical density), each image was ranked based on its relative ability to consistently generate changes in optical density across multiple recording sites. From this preliminary analysis, a positive/negative-valence pairing was selected for the classification problem. At the beginning of the session, the participant viewed a self-paced slideshow of images to be presented and was instructed to familiarize himself/herself with each image's contents. The participant completed 6 practice trials to acquaint himself or herself with the task. He/she then performed 30 trials of the emotional induction task for each image of the positive/negative-valence pair in 10 6-trial blocks. Images were presented in randomized order. To alleviate fatigue, halfway through the session a 10-minute break was imposed where the participant was asked to vacate the testing area.

### Artifact removal

Concentration changes in oxygenated and deoxygenated hemoglobin, denoted respectively as Δ[HbO] and Δ[Hb], were calculated at each of the 8 recording sites from changes in detected light attenuation using the modified Beer-Lambert Law before undergoing artifact removal. The modified Beer-Lambert law states that changes in optical density (ΔOD) can be calculated from a measured change in light attenuation before and after a test condition:(1)

where *I*_*B *_and *I*_*A *_represent light intensity measured under mean baseline and activation conditions, respectively, for the problem of interest. ΔOD is proportional to the extinction coefficient for molar concentrations of the light-absorbing compound (ϵ), the concentration of the compound (*c*), and optical path length. The optical path length is expressed as a product of source-detector distance *r *and a multiplier known as the differential pathlength factor (*DPF*), which is a function of the extinction coefficient of the scattering medium [29].

Total changes in light attenuation are expressed as a linear sum of contributions from each absorbing compound. Since the primary absorbers of NIR light in cerebral tissue are HbO and Hb, (1) can be expanded as:(2)

where *OD*^*λ *^equals optical density at wavelength *λ*,  and  are the extinction coefficients for *HbO *and Hb at *λ*, and *DPF*^*λ *^is the differential pathlength factor for the adult human head at *λ*. It follows that Δ[HbO] and Δ[Hb] can be determined by calculating changes in optical density at two wavelengths, *λ*_1 _and *λ*_2_. Solving the system of equations obtains Δ[HbO] and Δ[Hb]:(3)

We used literature values for *DPF *[29] and ϵ at the relevant wavelengths [30] to calculate Δ[HbO] and Δ[Hb]. At a sampling rate of 31.25 Hz, 1875 delta concentration values were obtained for each of HbO and Hb during one 60 s trial of the emotional induction task.

Adaptive noise cancellation has been shown to be effective in removing artifacts from EEG and fMRI brain recordings [31,32]. Some research groups have employed the technique to remove physiological artifacts from NIRS recordings [33,34]. We used a least-mean squares (LMS) adaptive filter to remove respiratory artifacts from the hemodynamic signals. Each respiratory signal was first resampled at 31.25 Hz and synchronized to its corresponding hemodynamic signal via a b-spline curve registration procedure [35]. We implemented landmark-based registration based on the alignment of local maxima and minima found in each pair of signals. To facilitate landmark estimation in the hemodynamic signal, signal components over the frequency range of interest were isolated; as such, Δ[HbO] and Δ[Hb] signals were filtered using a 0.4-1 Hz bandpass filter prior to registration. The respiratory signal was then registered to the filtered hemodynamic signal. An adaptive filter with 200 taps was used, and the step size was set to 0.001. Both values were empirically determined. It was noted that at a 31.25 Hz sampling rate 200 taps corresponds to 6.4 s (approximately 2 breaths), which is sufficiently long for modelling the characteristics of the respiratory signal.

Systemic low-frequency oscillations in the hemodynamic signal believed to arise from regional cerebral blood flow [36] are centered around 0.1 Hz [37]. We filtered out these vasomotion effects using a 3rd order Butterworth filter with a 0.05-0.15 Hz passband. Arterial pulsatility due to systole and diastole are visibly manifested as a series of periodic spikes superimposed over the slowly evolving hemodynamic response. A 30-point moving average filter, which corresponds to data spanning over approximately 1 s, was applied to reduce cardiac effects prior to feature extraction.

### Feature selection and classification

Δ[HbO] and Δ[Hb] signals were segmented into baseline and activation intervals to form two sets of 60 (30 baseline, 30 activation) trials for each stimulus. The transition point between the baseline and activation intervals was set as the time of initial stimulus presentation. Six time-domain and seven time-frequency domain features for classification were calculated for Δ[HbO] and Δ[Hb] signals for each trial over each recording site:

1. Mean: average signal value.

2. Variance: measure of signal spread.

3. ZC: Zero Crossings; number of instances where the signal crossed the zero line.

4. RMS: Root Mean Squared; measure of average signal magnitude.

5. Skewness: measure of the asymmetry of signal values around its mean relative to a normal distribution.

6. Kurtosis: measure of the degree of peakedness of a distribution of signal values relative to a normal distribution.

7. E_*a*_: percentage of total signal energy contributed by the approximation signal from a 6-level wavelet decomposition (Daubechies 4) of the time-domain signal.

8. E_*dX*_: percentage total signal energy contributed by each detail signal from a 6-level wavelet decomposition (Daubechies 4) of the time-domain signal. Six percentages were extracted, one for each level of decomposition (X = 1,...,6). Given the length of the signal input, the nominal maximum number of levels for a wavelet decomposition using a Daubechies 4 wavelet is six.

208 candidate features (13 features × 2 signals × 8 sites) were thus calculated for each participant. Research groups to date have primarily focused on classifying NIRS data using mean changes in hemoglobin concentration as a discriminatory feature [20,21]. In the present study, a large number of candidate features were introduced to the classification problem in an attempt to better characterize the space of possible features (i.e. search space), which contains a number of irrelevant or redundant features for classification. Feature subsets were selected for the classification task. Given the number of trials collected (60), only a two-dimensional feature space was justified. Feature selection was conducted for each participant using all combinations of the following performance parameters for each of the two classifiers of interest:

1. Task Valence (Positive/Negative): We hypothesized that classification performance correlates positively with subjective evaluation of task difficulty. If a participant finds it easier to perform one of the emotional induction tasks over the other - that is, associate emotions more strongly with one of the visual cues in the pairing - the data from the task may yield higher classification rates.

2. Recording Sites (Right Prefrontal/Left Prefrontal): We hypothesized that task valence correlates with optimal recording site according to the valence hypothesis, which posits that positive emotions are left-lateralized and that negative emotions are right-lateralized [38].

3. Analysis interval (15 s/20 s): We hypothesized that the optimal analysis interval is feature-dependent. We selected time intervals over which signal differences between baseline and activation states were expected to be observed given that the hemodynamic response peaks about 10 s from the start of the task [13,14]. Therefore, we compared classifier performance using features calculated over analysis time intervals of 15 s and 20 s.

All combinations of classifiers, task valences, recording sites, and analysis interval lengths generated 16 possible feature selection problems.

When appropriately configured, random search algorithms such as genetic algorithms (GAs) allow for the evaluation of a search space more efficiently than most other heuristic search methods [39] and perform well on noisy search spaces containing local minima [40]. Feature selection was thus performed using a standard GA with a rank-based parent selection strategy, a scattered crossover operator, and a uniform mutation operator (Genetic Algorithm and Direct Search Toolbox, MATLAB).

For each of the 16 problems, 20 runs of the GA were performed with the following parameter settings: 1) population size = 100; 2) number of generations = 30; 3) probability of crossover = 0.6; and 4) probability of mutation = 0.01. Parameter values were selected on the basis of results from several preliminary runs, and align with typical values used in literature [41]. We selected the feature set most frequently converged upon by the GA across the 20 runs. In the event of a tie, the feature set with the higher mean fitness value was selected. The fitness value of each candidate feature subset was defined by its 5-fold cross-validation classification accuracy. A Gaussian radial basis function kernel with unity scaling factor and penalty term was selected for the SVM classifier (Bioinformatics Toolbox, MATLAB).

Ten (10) runs of 5-fold cross-validation were then performed using the optimal feature set selected for each of the 16 problems. Fifty (50) accuracy measures (classification rates) were obtained after 10 runs of 5-fold cross-validation, from which a mean classification rate was calculated. We report the maximum classification rate obtained for each participant, along with corresponding feature set and performance parameter settings.

### Quantifying response latency

Classification accuracy was used to quantify when changes from a baseline state can be detected. Using the optimal feature set for each participant, mean classification rates were calculated via 10 runs of 5-fold cross-validation, over a range of analysis interval lengths. The baseline rate was arbitrarily defined as the mean classification accuracy calculated with an analysis interval of size Δ*T *= 1.0 s. The size of the interval was increased in 0.1 s increments from the transition point to a maximum of Δ*T *= 20.0 s. The minimum analysis interval length was set based on the number of points required for a 1-level wavelet decomposition using a Daubechies 4 wavelet.

**Figure 3:**
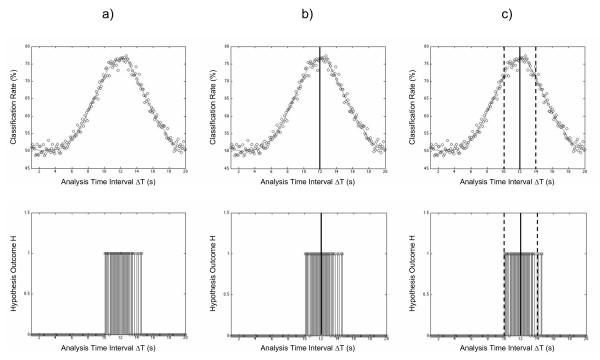
**Quantifying response latency**. Quantifying response latency. (a) Representative plots of classification rate vs. analysis time interval (top) and hypothesis outcome (H = 1 denotes significant difference from baseline rate) vs. analysis time interval (bottom). (b) Maximum mean classification rate is identified by a solid line. (c) Range of analysis intervals with significant activation demarcated by the dashed lines.

Next, we checked for statistically significant differences between the set of classification accuracies calculated at Δ*T *= 1.0 s and each set of classification accuracies calculated at Δ*T *= (1.0 + *t*) s, where *t *ranged from 0.1 to 19.0. These results were used to determine a range of analysis interval lengths over which statistically significant activation was detected (Fig. 3):

1. Mean classification accuracy was plotted as a function of analysis interval size. The accuracies were loess smoothed using a span equal to 20% of the number of data points. Hypothesis test outcome H was also plotted as a function of analysis interval size. H(Δ*T*) = 1 indicates that a statistically significant difference from baseline accuracy (*p *< 0.05, corrected resampled t-test) was detected at analysis interval Δ*T*.

2. The vector of smoothed accuracies was searched for its maximum value (i.e. maximum classification rate), and its corresponding analysis interval length (Δ*T*_*max*_) was noted.

3. To quantify the range of analysis interval lengths with statistically significant activation, two iterative searches were performed forwards and backwards from Δ*T*_*max*_. The mean classification rate at Δ*T *= *v *s (0.1 ≤ *v *≤ 20) was deemed significantly different from the baseline rate if *H *= 1 for > 50% of the original (unsmoothed) data points in the range Δ*T *= *v *± 0.5 s. A search was terminated when the aforementioned condition was violated and the termination point marked as a boundary of the range of analysis interval lengths with significant activation.

## Results

### Feature selection

The feature set and combination of performance parameters that yielded the highest mean classification accuracy for each participant were identified. Table 1 summarizes the results for GA-based feature selection. Features were selected across a range of recording sites, which is not entirely unexpected given NIRS' limited spatial sensitivity. Though [Hb] is thought to be a more reliable indicator of functional activation [42], the GA selected features derived from Δ[HbO] and Δ[Hb] signals with equal frequency. This implies that among other physiological phenomenon, Δ[HbO] captures valuable information directly correlated with experimentally derived activations and should not be discarded.

**Table 1:** Results for GA-based feature selection.

**Participant No**.	Common features Selected Across **Performance Parameter Sets**^**1**^	Optimal Parameter Set		
		
		**Symbol**^**2**^	Feature Pair	**Classification Accuracy**^**3**^
1	Mean, Skewness	LDA-L-20-	MeanHbO_*L*1_ MeanHbO_*L*4_	75.00 ± 10.83%
2	Mean, Skewness	LDA-L-20+	MeanHbO_*L*3_ MeanHbO_*L*4_	89.67 ± 7.82%
3	Mean, Skewness	LDA-L-20+	MeanHbO_*L*1_ MeanHbO_*L*4_	96.67 ± 5.32%
4	Kurtosis, Skewness	LDA-L-15-	KurtosisHbO_*L*4_ SkewnessHbO_*L*3_	75.33 ± 12.59%
5	Kurtosis, Skewness	LDA-L-15-	KurtosisHbO_*L*3_ SkewnessHb_*L*2_	88.00 ± 7.93%
6	Kurtosis, Skewness	SVM-L-20-	SkewnessHbO_*L*1_ SkewnessHbO_*L*2_	75.83 ± 10.55%
7	Mean	SVM-L-20+	MeanHb_*L*4_ VarianceHb_*L*2_	94.67 ± 5.77%
8	Mean, Skewness, E_*a*6_	LDA-R-20+	MeanHb_*R*3_ ZCHbO_*R*3_	89.00 ± 8.82%
9	Mean, Skewness	LDA-R-15+	EaHb_*R*3_ SkewnessHb_*R*3_	83.83 ± 9.88%
10	Mean, Skewness, E_*a*_	LDA-R-20+	Ed6HbO_*R*3_ MeanHbO_*R*3_	78.00 ± 9.78%

^1^Found in ≥25% feature pairs across performance parameter sets

^2^Symbol defining classification scheme consists of 4 parts: Classifier (LDA/SVM) - Recording Side (L/R) - Analysis Time Interval (15/20) - Stimulus Valence (+/-)

^3^10 randomized trials, 5-fold cross-validation

Regardless of the classifier of interest, time-domain features, i.e. either one of skewness or mean of Δ[HbO] and Δ[Hb], were consistently selected by the GA as part of the optimal feature pair across and within participants. The aforementioned time-domain features were frequently selected for each participant across the 16 feature selection problems. The GA occasionally selected time-frequency features, and even then, only alongside a time domain feature; it thus appears that time frequency features merely provided information that supplemented the discriminatory time domain features. Time-domain features alone may be sufficient for online implementation of a NIRS corporeal machine interface.

No performance parameters had a significant effect on inter-subject classification accuracy. Average accuracies did not differ between LDA and SVM classifiers (p ≥ 0.05, corrected resampled t-test [43]). Interestingly, optimal classification accuracy was achieved for 8 of the 10 participants with an LDA-trained classifier, which is advantageous for its computational speed and ease of implementation.

Results indicate that the optimal analysis time-scale varies with the choice of signal features. A 20 s analysis interval was selected for all participants classified using a 2-feature vector containing at least one feature representing signal mean. Discriminatory information may be present in the NIRS hemodynamic signal for a prolonged period after its peak latency since the hemodynamic response needs more than 10 s to return to baseline [44,45]. In contrast, a 15 s analysis interval was selected for 3 of 4 participants classified using signal skewness as a primary feature.

### Classification

**Figure 4:**
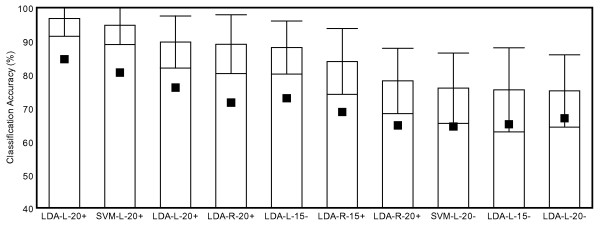
**Classification results across participants ranked by accuracy**. Classification results across participants ranked by accuracy. Black squares denote lowest accuracy obtained across 16 feature selection problems. X-axis labels indicate optimal feature set (label defining optimal feature set consists of 4 parts: Classifier - Recording Side - Analysis Time Interval - Stimulus Valence). Error bars denote standard deviation.

Maximum percent correct classification (*PCC*_*max*_) rates across participants ranged from 75.0%-96.7%. Several trends become apparent after participant results were ranked by accuracy (Fig. 4). The four highest classification accuracies were produced using mean changes in [HbO] and [Hb] as discriminatory features. Additionally, six of the top seven performers achieved optimal accuracy in response to positively-valenced stimuli. This suggests that the time course of hemodynamic activity generated by emotional induction tasks may be influenced by valence.

**Figure 5:**
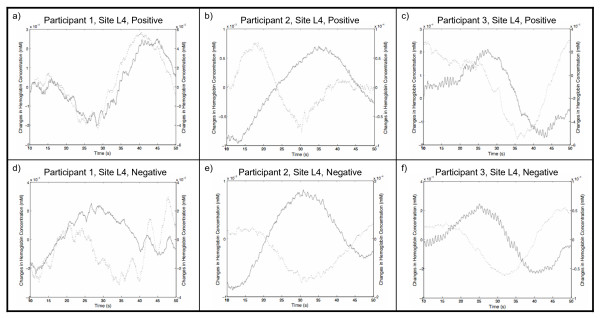
**Trial-averaged Δ[HbO] and Δ[Hb] data from Participants 1 - 3**. Trial-averaged Δ[HbO] (red) and Δ[Hb] (blue) data over t = 10 - 50 s from Participants 1 - 3 performing positively and negatively-valenced emotional induction tasks. Note different coupling trends between and within participants.

A comparison across participants provided insight into why classification rates may vary. Figure 5 illustrates the trial-averaged hemodynamic response at site L4 for Participants 1 through 3. The GA selected a common feature (*MeanHbO*_*L*4_) and identical parameters (classifier, recording sites, analysis interval length) for all three individuals. Participants 1 and 3 shared identical features and parameters with the exception of stimulus valence, and achieved the lowest and highest classification accuracies, respectively.

Participant 3 (*PCC*_*max *_= 96.67%) generated a consistent response using both valenced stimuli. A decrease in Δ[HbO] was observed for the duration of the emotional induction task (t = 30 - 40 s), which corroborates with previous study findings on sustained attention [17]. We see a small increase in Δ[Hb] shortly after stimulus presentation consistent with the temporal profile of the NIRS hemodynamic response [15]. These trends were also present in Participant 2's data (*PCC*_*max *_= 89.67%), although there is a longer latency before Δ[HbO] ceases to decrease. In the case of Participant 1 ((*PCC*_*max *_= 75.00%), hemodynamic activity was only visible in the signals generated by the negatively-valenced task. The trial-averaged Δ[HbO] and Δ[Hb] signals also contained larger fluctuations that obfuscated longer time-scale trends. Combining the findings described above, we propose that classification rates are limited by: 1) one's ability to consistently perform the emotional induction task; and 2) the hemodynamic response's rate of change.

### Response latency

**Figure 6:**
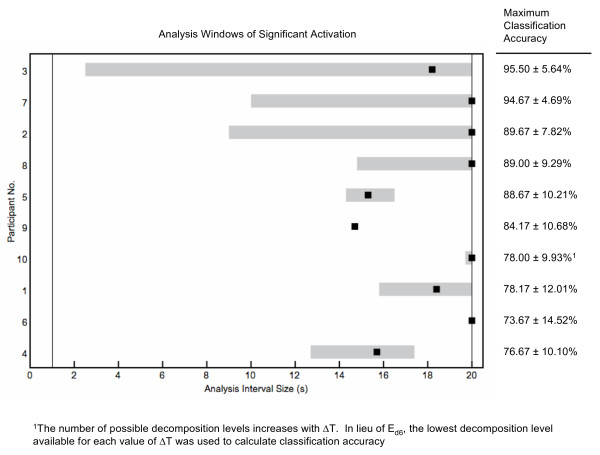
**Response latency analysis results across participants ranked by classification accuracy**. Response latency analysis results across participants ranked by classification accuracy. Range of analysis interval sizes (Δ*T*) where statistically significant increases in classification rates were detected from baseline classification rates is indicated in gray. Δ*T*_*max*_, the analysis interval size corresponding to *PCC*_*max*_, is indicated as a black square.

From visual inspection of trial-averaged hemodynamic signals, it is apparent that response latency varies among individuals. Figure 6 summarizes optimal analysis interval lengths across participants. Each horizontal bar represents the analysis interval range for which significant activation was detected for a participant.

We begin by defining values of interest: 1) Δ*T*_*start*_, the smallest value of Δ*T *for which significant activation is detected; 2) Δ*T*_*max*_, the value of Δ*T *corresponding to *PCC*_*max *_over all analysis interval lengths tested; and 3) Δ*T*_*end*_, the largest value of Δ*T *for which significant activation is detected. Δ*T*_*start *_and Δ*T*_*end *_define the activation window.

The average time for onset of activation was 12.4 s across participants for whom significant activation was detected. Significant activation was not detected for Participants 6 and 9 and hence their data are not included in this average. It was earlier noted that the optimal feature pair selected for each participant included one of skewness or mean, which we define as a "primary discriminatory feature". Activation windows can be characterized by the primary discriminatory feature employed for classification:

• *Mean *(*n *= 6) Classification rates improved with increased Δ*T*. Δ*T*_*max *_for all individuals was 20.0 s, the largest interval size considered in our analysis. These observations agree with results from the feature selection procedure. Participants with higher classification rates had shorter onset times prior to significant activation. Values of Δ*T*_*start *_varied but generally exhibited an inverse relationship with *PCC*_*max*_, ranging from 2.5 s (Participant 7, *PCC*_*max *_= 95.50%) to 19.7 s (Participant 10, *PCC*_*max *_= 78.00%).

• *Skewness *(*n *= 4) Classification rates also improved with Δ*T *but peaked before Δ*T *reached 20.0 s. With the exception of one individual - for whom significant activation was not detected - Δ*T*_*max *_ranged from 14.7 s to 15.7 s. This suggests an analysis interval of Δ*T *= 15 s is nearly optimal for a feature set that includes skewness. For each of these participants, we identified a short range of analysis interval lengths surrounding Δ*T*_*max *_where significant activation was detected. Activation window sizes ranged from 0.0 s to 4.7 s. Differences between *PCC*_*max *_and baseline rates did not reach significance.

## Discussion

We have established that distinct patterns of hemodynamic activity generated by a visually-cued emotional induction task can be detected using NIRS and classified offline with accuracies significantly exceeding chance levels. Classification rates were comparable with values reported in previous NIRS-BCI studies. Six of the ten participants reached mean classification rates that exceeded the 70% threshold (p < 0.05) suggested by the scientific community as sufficient for communication and device control [46]. It is conceivable that this number may have been higher if more trials were collected; however, data set size was inherently limited by the repetitive nature of the protocol and the mental demand of the task on the participant.

The onset time for a detectable hemodynamic response varied across individuals. Regardless of the types of features used for the classification task, a significant increase in mean classification accuracy was detected for the majority of participants 10 - 15 s after presentation of the visual stimulus. These latencies are in line with values previously reported in NIRS literature [13,14].

### Neurological and psychological factors

Participants generally found the emotional induction task straightforward to perform, and based on the experiences drawn from their involvement in the study, felt that such a paradigm can potentially be implemented in a user-friendly online corporeal machine interface.

Nevertheless, there are several factors that likely impacted data consistency within and across participants. Despite implementing preventative measures in the protocol to mitigate fatigue, four participants cited various aspects of the study as physically tiring. Incorporation of on-line feedback into the experiment may help maintain the participant's concentration and improve performance by providing a clear goal to the task. While one can argue that the benefits of neurofeedback are negligible over a single session, neurofeedback training is essential for operant conditioning of the EEG [47] and fMRI-BOLD responses [7,8]. A participant may also begin the emotional induction task at a different time for each trial, further contributing to data inconsistencies. Possible causes include anticipatory effects [48] and loss of focus due to fatigue [49].

Some participants found the task easier to perform over time, whereas others found it increasingly difficult to concentrate as he or she repeatedly viewed the same pair of images. This may be attributed to the unique mental strategy each individual cultivated for performing the emotional induction task. Individual strategies ranged from using the image as a visual cue to focus on a more general emotion, to focusing on a salient component in the image. The PFC is involved in maintaining attentional demand [50], and variations in intensity and latency of hemodynamic activity across individuals might be caused by the different levels of attentional demand required for different strategies. Another possible explanation is that each participant's response to a stimulus was motivated by a different variation of endogenous salience, thereby eliciting different patterns of PFC activity. The image either functioned as a "primary inducer" conveying some intrinsic value, or acted as a secondary inducer that triggered the recall of a related memory or event [51]. The latter, commonly referred to as self-referential processing [52], is accompanied by a more intense emotional response provided the stimulus contains personal relevance. Notably, the medial prefrontal cortex (mPFC) has been implicated in self-referential processing [52]; however, because participants were not provided with specific instructions on how to perform the emotional induction task, we cannot draw conclusive inferences about mPFC activity and self-referential processing.

The fact that results from feature selection did not suggest a correlation between stimulus valence and lateralization of brain activity may be due to optode placement. Optodes were located more medially than in several neuroimaging studies on emotional processing that have reported hemispheric specialization in the lateral PFC [53,54]. In a metaanalysis of emotional activation studies, it was found that the mPFC is systematically activated by emotional stimuli regardless of valence [55]. This suggests that the mPFC plays a general, rather than specific, role in emotional processing primarily mediated by arousal. It corroborates with our observation that a participant generally achieved higher classification rates using a stimulus he/she subjectively perceived as being more emotionally arousing. Five out of six participants who stated a preference for one image in the positive-negative valence picture pair achieved optimal classification accuracy using his or her preferred stimulus. To ascertain the effects of self-relevance in future studies, it would be beneficial to incorporate self-assessment of valence/arousal by each participant for each image.

### Anatomical considerations

Because the NIRS method measures venous, arterial and tissue oxygenation, it is more sensitive to localized concentration changes in skin microvasculature than underlying tissue volumes [56]. Combined with the choice of a short source-to-detector separation distance, one may argue that our findings are based solely on oxygenation saturation of the extracranial layer, and are not indicative of functional activation in the cortex. We disagree that this is a limitation of the protocol. In a subset of study participants, we confirmed that hemoglobin concentration changes detected over a 2.1 cm spacing are highly correlated with adjacent measurements acquired over a 3.0 cm spacing, which is commonly used in fNIRS studies. It could also be argued that given the objectives of our study, the physiological origin of the detected hemodynamic response is secondary in importance to the ability to consistently generate a response.

Furthermore, scalp and skull thicknesses vary around the head of an individual [57]. This contributes to variations in signal strength over different recording sites, and is a possible reason why we did not observe any trends in the site locations selected by the GA. The thickness of the extracranial layer dictates the minimum source to detector separation required to probe the cerebral cortex, and ideally, would be optimized for each individual. These dimensions are unknown unless an MRI scan is procured.

### Limitations

Although NIRS offers advantages over conventional EEG interfaces, it introduces instrumentation challenges unique to the technology. Hemodynamic signals are resistant to motion artifacts provided that optodes can be mounted firmly to the skin. However, it is a non-trivial task to secure optical fibres to the head, and design solutions must achieve a balance between stability of the optical fibres, versatility to accommodate a range of head sizes, and comfort. New methods are continuously being developed and a number of solutions have been implemented to date [58]. Secondly, melanin is a known source of attenuation for optical throughput over the NIR range [58]. While absorption and coupling issues caused by hair can be circumvented by measuring over hair-free regions such as the forehead, signal strength and penetration depth remain affected by skin colour.

The long latency of the hemodynamic response severely limits the information transfer rate of a NIRS corporeal machine interface. However, in addition to the hemodynamic response, frequency-domain NIRS measurements may yield a second "fast optical response" directly correlated with neuronal firing. The fast optical response is believed to be caused by changes in light scattering properties of neuronal membranes synonymous with activated cerebral tissue [15] and is elicited milliseconds after tissue stimulation [10]. Not all researchers are convinced that the fast optical response can be detected non-invasively owing to the fact that the signal is dominated by other physiological artifacts [36,59], and simulation results suggest that the magnitude of the fast optical response is below the noise level of presently available NIRS systems [60]. If commercial systems that reliably capture the fast optical response become available, NIRS corporeal machine interfaces that respond as quickly as conventional EEG interfaces can be developed.

A priori knowledge of the latency of the hemodynamic response, which has been shown to vary across individuals, may be used to address the above shortcoming. For instance, if the optimal parameters and analysis interval length for signal classification were known for a user, the knowledge can be utilized to customize a corporeal machine interface, thus maximizing his or her abilities and improving response times. Since we could only collect a limited number of trials per participant within an experimental session, we did not have sufficient sample sizes to create completely disjoint data subsets for feature selection and classifier development. While our results may be therefore be optimistic, i.e., akin to "training accuracies", they are nonetheless on par with those reported for NIRS-BCIs using different mental tasks. Practically, a long data collection session (> 2 hours) only yields a modestly-sized data set per participant, given the non-trivial time periods for the cyclic generation and dissipation of hemodynamic responses. Thus collecting large data sets, while necessary, will remain a practical challenge for NIRS-based corporeal machine interfaces in future studies.

In our analyses, we attempted to suppress non-cortical contributions by low pass iltering. This is an inherent limitation as we do not have direct knowledge of the contaminant frequencies. Therefore, the signals we have classified are inevitably comprised of a combination of cortical and systemic blood flow. Other studies have suggested the simultaneous acquisition of deep and shallow signals using an optode arrangement consisting of multiple source-detector separations [61,62]. In this way, systemic effects recorded in the shallow signal can be directly attenuated in the deep (cortical) signal.

### Future directions

The reliability of the proposed paradigm should be verified with simultaneous acquisition of fMRI and NIRS data, which would allow for accurate localization of externally recorded signals with respect to underlying anatomy. Qualitative amplitude correspondence of NIRS signals to the fMRI-BOLD response can provide insight into which types of emotional induction tasks are best suited for corporeal machine interfaces and their underlying psychophysiological bases.

As an extension to user customization in corporeal machine interfaces, it would also be desirable to investigate the effects of varying the time window for visual stimulus presentation. Like the analysis interval length for signal classification, this parameter could conceivably be optimized such that hemodynamic activity is generated reliably with less effort. Additional types of stimuli for the emotion induction paradigm should be investigated. Somatosensory and auditory stimuli are suitable alternatives for those with visual deficits, as well as multimedia stimuli such as film or music.

## Conclusion

This study ascertained the feasibility of NIRS as a platform for a corporeal machine interface. We demonstrated that an emotional induction task in neurologically healthy individuals can elicit measurable hemodynamic responses in the prefrontal cortex. Classification accuracies up to 96.7% were obtained after feature subset selection while varying several performance parameters of interest. Results from the feature selection procedure indicate that mean and skewness parameters are the best discriminatory measures between resting and activation states induced by our task of interest. Relationships were also identified between a number of parameters, namely, feature subset and analysis interval length, and stimulus valence and classification accuracy. Lastly, classification accuracy was used to quantify the latency of the hemodynamic response within participants, with significant increases in accuracy from baseline occurring as early as 2.5 s from initial presentation of the stimulus.

## Competing interests

The authors declare that they have no competing interests.

## Authors' contributions

KT conceptualized the study, carried out the data collection procedure, conducted data analyses, and drafted the manuscript. TC contributed to study conception, supervised the study and revised the manuscript. All authors read and approved the final manuscript.
